# A Case of Cesarean Operation Performed with Success

**Published:** 1859-09

**Authors:** 


*A case of Cesarean Operation performed with success. By M. Borie.* (Memoires de l' Academie Imperiale de Medicine. Paris, 1857-58.)

"This was performed at the Maternite at Tulle, on the person of a ricketty but hardy primipara, aged 29, in whom the space between the sacro-vertebral angle and the triangular ligament of the symphisis pubis measured but from five to six centimetres at the utmost. A living child was delivered, and the woman recovered rapidly. Chloroform was employed, and a longer incision than usual was practised in opening the cavity of the abdomen."—*Medico-Chir. Rev., July,* 1859.

